# Timing of recurrences of TEM resected rectal neoplasms is variable as per the surveillance practices of one tertiary care institution

**DOI:** 10.1038/s41598-021-85885-0

**Published:** 2021-03-22

**Authors:** Allison C. Keeping, Paul M. Johnson, Christopher R. Kenyon, Katerina Neumann

**Affiliations:** Division of General Surgery, Dalhousie University, Halifax, NS B3H 2Y9 Canada

**Keywords:** Rectal cancer, Colonoscopy

## Abstract

Transanal endoscopic microsurgery (TEM) is widely used for the excision of rectal adenomas and early rectal adenocarcinoma. Few recommendations currently exist for surveillance of lesions excised by TEM. The purpose of this study was to review the surveillance practices and the patterns of recurrence among TEM resected lesions at a tertiary care hospital. A retrospective chart review was performed on all patients who underwent TEM for rectal adenoma or adenocarcinoma before June 2017. In our study population of 114 patients, the final pathology included 78 (68%) adenomas and 36 (32%) adenocarcinomas. Of the adenocarcinomas 23, 9, and 4 were T1, T2, T3 lesions, respectively. Of those, 25 patients opted for surveillance instead of further treatment. The most commonly recommended endoscopic surveillance strategy by our group for both adenomas and adenocarcinomas excised by TEM was flexible sigmoidoscopy every 6 months for 2 years. Recurrences occurred in 4/78 (5.1%) adenoma patients, all found within 16.9 months of surgery, and in 4/25 (16%) adenocarcinoma patients, found between 7.4 and 38.5 months post-surgery. Our data highlights the fact that the timing of recurrences post TEM surgery is variable. Further studies looking at recurrence patterns are needed in order to create comprehensive guidelines for surveillance of these patients.

## Introduction

Colorectal cancer represents a significant contributor to disease burden in Canada and worldwide. It is the second leading cause of cancer deaths in men and third leading cause in women, representing 12% of all cancer deaths according to the Canadian Cancer Registry^1^. The incidence of colorectal adenoma, the precursor lesion of colorectal cancer, is very high worldwide. It is estimated that 20–53% of the American population over the age of 50 have adenomas of the colon^2^. Many of these cases go undetected. Those that are diagnosed, can often be removed during endoscopy. In some cases, adenomas are too large to safely remove using endoscopic means thereby requiring surgery.

Traditionally, benign lesions of the rectum have been removed either by local excision or—if located in the mid-upper rectum and out of reach of the standard proctoscope—with radical resection (RR). Transanal endoscopic microsurgery (TEM) has offered an alternative option for the management of such lesions since its introduction in 1988 by Dr. Gerhard Buess, as it allows access up to 20 cm from the anal verge for high quality full-thickness local excisions^3^. Studies have shown that TEM achieves lower risk of positive margins and lower recurrence rates over traditional transanal excision for rectal adenomas^4^. Recurrence rates range from 2 to 16% after TEM excision compared to 28–32% after traditional transanal excision of rectal adenomas^5,6^, making TEM the superior modality of excision. Many have stratified adenomas by size, classifying giant adenomas as those lesions greater than a given minimal size ranging between 3 and 5cm^7–9^. McCloud showed that giant polyps > 5 cm are associated with higher local recurrence rates of 25.9% in comparison to smaller sized polyps after excision by TEM^10^.

Several studies have shown that T1 rectal adenocarcinoma can also be safely removed by TEM, with similar overall survival, reduced morbidity and shorter hospital stay compared with RR^11–13^, as well as low recurrence rates, most studies ranging from 0 to 13%^5,6,10^. This is still slightly higher than the risk of local recurrence with RR (0–2%) when treating T1 cancer, although many find this an acceptable risk weighed against the benefit of less invasive surgery. Radical resection continues to be the preferred treatment for T2 cancers and beyond^11,12^.

Since TEM is associated with a higher rate of recurrence for T1 rectal cancer than radical resection adequate post-operative surveillance is especially important. This is also true for many TEM-excised benign polyps because the lesions requiring TEM are often quite large thus also at increased risk of recurrence. Currently, there are no established guidelines for the post-operative endoscopic surveillance of patients after TEM. When it comes to benign disease, it is unclear whether one should follow the standard post-polypectomy guidelines, or whether to scope patients more frequently. Canadian clinical practice guidelines exist regarding surveillance for patients with T2/T3 colorectal cancer following RR, but not yet for T1 rectal cancers excised locally by TEM or otherwise^14,15^. The American Society of Colon and Rectal Surgeons clinical practice guidelines state that “patients with T1 rectal cancer treated with local excision should be considered for surveillance, although the evidence for its utility is conflicting”^16^.

This case series aims to evaluate the post-operative surveillance practices of the surgeons performing TEM at a tertiary care teaching hospital, and to determine the patterns of recurrence among the adenoma and adenocarcinoma patient populations.

## Methods

All methods were approved by the Nova Scotia Health Authority Research Ethics Board and completed in accordance with applicable guidelines and regulations including patient privacy and confidentiality regulations. Informed consent of participants was not required as documented in the Waiver of Consent approved by the Nova Scotia Health Authority Research Ethics Board. All patients undergoing TEM at a tertiary care teaching hospital between January 2012 and June 2017 were identified. Patients with a post-operative histology confirmed diagnosis of adenoma or adenocarcinoma were included in this analysis. Excision of scar was included, even if no residual lesion was found on the final pathology of the TEM specimen, as long as the histology on the polypectomy specimen confirmed the presence of adenoma or adenocarcinoma. TEM for recurrent adenoma after previous traditional local excision was included. Repeat TEM surgeries for recurrences after previous TEM were not included but were documented as part of the treatment plan following the original index surgery. Patients who underwent TEM for palliation were excluded, as well as TEM cases that were aborted prior to attempt at dissection.

Data collected included patient demographics, endoscopic location, and height of tumour and histopathology from endoscopic biopsy. Height of tumour was determined by rigid sigmoidoscopy or flexible sigmoidoscopy measurement from anal verge, when available, in that order of preference. If no measurements were available, data on height was extracted from imaging. Operative data included length of surgery, surgeon, patient position, intraoperative complications, whether the defect was closed, histopathology and margins. Post-operative information collected included length of stay in hospital, short and long-term complications, and if salvage surgery was required, as well as any adjunct treatments. Surveillance clinic and endoscopic visits were recorded. Data was collected on timing of recurrences and subsequent treatment plan. All available endoscopic follow up visits at the time of data collection were recorded. Disease specific and overall mortality was recorded.

## Results

One hundred and nineteen patients were identified that underwent TEM during the specified time, of which 5 were excluded. Two patients were excluded who underwent TEM for palliative reasons; 2 had diagnosis other than adenoma or adenocarcinoma; 1 patient was excluded because surgery was aborted prior to attempt at dissection. For the remaining 114 patients demographics, tumour factors, operative details and pathology are shown in Table 1. The final pathology included 78 (68%) adenomas and 36 (32%) adenocarcinomas. Post-operative complications are shown in Table 2. Median follow-up was 11.7 months with a range from 2.5 to 54.4 months. Post-operative endoscopic follow up data was available for 74 (64.9%) of the patients.

**Table 1:** Patient demographics, tumour factors and operative/post-operative results.

Demographics	All Patients n = 114	Adenoma n = 78	Adenocarcinoma n = 36
Age, mean, [range]	67.4 [43–88]	66.5 [43–86]	69.2 [44–88]
Sex, male	68 (59.6%)	43 (55.1%)	25 (69.4%)
BMI, mean [range]	28.5 [19.1–49.4]	28.7 [19.7–49.4]	28.1 [19.1–36.1]
**ASA**			
I	23 (20.2%)	18 (23.1%)	5 (13.9%)
II	70 (61.4%)	47 (60.3%)	23 (63.9%)
III	21 (18.4%)	13 (16.7%)	8 (22.2%)
Mean Tumour Height* (cm), [range]	6.6 [2–18]	6.7 [2–18]	6.5 [2–17]
**Size of lesion**			
< 4 cm	54 (47.4%)	37 (47.4%)	17 (47.2%)
4–8 cm	39 (34.2%)	34 (43.6%)	6 (16.7%)
> 8 cm	5 (4.4%)	2 (2.6%)	2 (5.6%)
No residual disease	13 (11.4%)	2 (2.6%)	11 (30.6%)
Piecemeal excision (no size reported)	3 (2.6%)	3 (3.8%)	
Mean Length of surgery (minutes), [range]	103 [27–417]	99.5 [39–417]	112 [27–187]
Depth of Dissection: full thickness	95 (83.3%)	61 (78.2%)	34 (94.4%)
Closure of defect	92 (80.7%)	61 (78.2%)	31 (86.1%)
**Margin status**			
Positive	18 (15.7%)	13 (16.7%)	5 (13.9%)
within 1 mm	7 (6.1%)	7 (9.0%)	0
**Dysplasia (n = 78)**			
High grade	–	28 (35.9%)	–
Low grade	–	9 (11.5%)	–
No Dysplasia	–	39 (50.0%)	–
Not reported	–	2 (2.6%)	–
**T Stage (n = 36)**			
T1	–	–	12 (33.3%)
T2	–	–	9 (25%)
T3	–	–	4 (11.1%)
Malignant Polypectomy scar	–	–	11 (30.6%)
**Sm levels for T1 lesions (n = 12)**			
sm1	–	–	8 (66.7%)
sm2	–	–	0
sm3	–	–	3 (25.0%)
Not reported	–	–	1 (8.3%)
Salvage RR after TEM	9 (7.9%)	0	9 (25%)

*data on tumour height was only available in n = 88 patients.

**Table 2:** Post-operative complications following TEM.

	All Patients (n = 114)	Adenoma (n = 78)	Adenocarcinoma (n = 36)
Bleeding	16 (14.0%)	13 (16.7%)	3 (8.3%)
Urinary Retention	14 (12.3%)	8 (10.3%)	6 (16.7%)
Fever	10 (8.8%)	5 (6.4%)	5 (13.9%)
Pelvic Pain	16 (14.0%)	10 (12.8%)	6 (16.7%)

For benign neoplasms, the planned endoscopic surveillance regimen quoted most often in the clinic notes was flexible sigmoidoscopy every 6 months for 2 years. For patients who chose surveillance after TEM for malignant neoplasms, the endoscopic surveillance regimen often included flexible sigmoidoscopy at 6 months, full colonoscopy at 12 months, flexible sigmoidoscopy at 18 months and 24 months. For some patients, the endoscopic regimen was not clearly outlined in the clinic notes upfront but appeared to follow this regimen. No alternative surveillance regimen was consistently documented in the clinic notes of our patient population. Of the 114 patients that underwent TEM, 74 had at least one surveillance endoscopy with the surgeon who performed their TEM. For the 40 patients who did not, the majority of them (31/40) had a clinic visit or phone call with the surgeon to discuss post-operative results, diagnosis, complications, and plan. Recommendations for further follow up was often documented in these encounters but the results of the endoscopy performed elsewhere were unavailable. There were 9 patients who did not have any documented post-operative follow up communication with the surgeon. The majority of the 74 patients who had post-operative endoscopic surveillance visits with the TEM surgeon, had them at the 6 month, 12 month, 18 month, and 24 month timeframes, respectively (± 3 months) (Table 3). Patients had a mean of 2.3 endoscopic visits with the TEM surgeon. The median number of months between the operating date and the respective surveillance endoscopy visits is reported in Table 3.

**Table 3:** Timing of Endoscopic Follow-up post TEM.

	6 months	12 months	18 months	24 months	> 27 months	Median time from OR to scope (mos)	Lost to follow up from first visit
First scope (n = 74)	61 (82.4%)	10 (13.5%)	2 (2.7%)	0	1 (1.3%)	6.5	n/a
Second scope (n = 45)	7 (15.5%)	28 (62.2%)	6 (13.3%)	3 (6.7%)	1 (2.2%)	12.3	29
Third scope (n = 28)	0	3 (10.7%)	17 (60.7%)	3 (10.7%)	4 (14.3%)	18.6	46
Fourth scope (n = 15)	0	1 (6.7%)	2 (13.3%)	8 (53.3%)	4 (26.7%)	25.1	59
Fifth scope (n = 10)	0	0	0	1 (10.0%)	9 (90%)	37.0	64

Endoscopy was recorded as meeting a timeframe if it occurred within 3 months of that timeframe.

Recurrences occurred in 4/78 adenoma patients (5.1%) and all were found by the second endoscopic follow-up visit or within 18 months of TEM surgery (Table 4). Only 1 of 4 recurrences had positive margins on the original TEM specimen. One was successfully treated by repeat TEM, one endoscopically, and one underwent radical resection. The fourth was treated by endoscopy in a palliative manner due to patient age and comorbidities.

**Table 4:** Summary of Adenoma recurrences.

Patient	Endoscopic Visit	Time to recurrence (months)	Size of original lesion (cm)	Original margin Status	Further Treatment
1	1	16.9	4.5	Negative	TEM
2	1	6.4	10.0	Negative	Polypectomy
3	2	10.4	5.5	Negative	RR
4	2	11.0	4.3	Positive	Palliation

Of 36 patients with a final diagnosis of adenocarcinoma, any patients with positive margins, high risk features, or > T1 depth of invasion were offered further treatment, with radical resection clearly depicted as the gold standard and adjuvant chemotherapy or radiation as alternative options. Those patients with T1, sm1 (submucosal level 1) pathology and negative margins were offered either surveillance or radical resection, after counselling by the surgeon about the recurrence rates with either of these approaches. Eleven of 36 patients with adenocarcinoma underwent radical resection or adjuvant therapy combined with radical resection immediately following the TEM surgery. In the remaining 25 adenocarcinoma patients who underwent surveillance only, recurrences occurred in 4 patients (16%) (Table 5). The recurrences were found between 7.4 and 38.5 months post TEM. Of the 4 patients who recurred, two underwent radical resection for treatment of their recurrence; the other two received palliative treatment. One of the recurrences was not detected until the 3^rd^ endoscopic surveillance visit at 38.7 months, after two normal endoscopies prior to 14.5 months.

**Table 5:** Summary of Adenocarcinoma recurrences.

Patient	Endoscopic visit	Time to recurrence (months)	Original margin status	Original T stage	Type of recurrence	Further treatment
1	3	38.5	Negative	T1 (sm1)	Local and Distant	Palliation
2	1	11.1	Negative	T2	Local	APR
3	2	7.4	Negative	T2	Local	Palliation
4	2	14.3	Negative	T1 (sm1)	Local	LAR

## Discussion

The focus of our study was on endoscopic surveillance practices post TEM and recurrence patterns in benign and malignant lesions, at our institution. The demographics of our patients, tumour characteristics, surgical results and outcomes are similar to other studies on TEM for benign and malignant lesions^17–22^. Although there is currently no established standardized protocol for endoscopic surveillance of patients post-TEM at our institution, the majority of the patients had their endoscopy within the timeframes of the commonly quoted surveillance recommendation from the clinic notes, namely every 6 months for 2 years.

In our benign adenoma population, recurrences occurred in 4/78 (5.1%) patients and were detected at the first or second post-operative endoscopic follow up, which ranged from 6.4 to 16.9 months from their original surgery date. Of note is the fact that our latest recurrence was caught at 16.9 months, however this patient did not have their first post-surgical endoscopic surveillance visit until 16.9 months, thus the recurrence may have been caught earlier had the patient been scoped at 6 and 12 months. Studies show similar recurrence rates ranging from 2.4%-10.5% as that seen in our adenoma patients^18,19^. A study by Barendse et al. showed that in benign lesions excised by TEM, larger size (> 3 cm) was a significant factor for recurrence and the median time to recurrence was 10 months^23^. Chan et al. had a slightly higher recurrence rate of 13.8% for benign lesions with a mean size of 4.3 cm however they had positive margins in 7.7% of cases^17^. They also demonstrated median time to recurrence of 387 days, or 12.9 months, and 80% of documented recurrences occurred by 40.8 months post TEM. Chan et al. recommend flexible sigmoidoscopy at 6 months, 2 years, and 3 years, along with full colonoscopy at 1- and 4-years post TEM, for TEM-resected adenomas^17^. There are few alternative recommendations in the literature currently. One could extrapolate from available post-polypectomy guidelines that as long as the TEM specimen is removed in one piece, pathology is benign with no high risk features, and margins are negative, that rigorous surveillance of the excision site would not be necessary, and a standard 3–5 year colonoscopy interval would be indicated^14,15,24,25^. However, we also know that TEM is selected for patients who have lesions that are unresectable by traditional polypectomy, either because of their size, polyp characteristics, or location. We feel that the available post-polypectomy endoscopic guidelines are therefore inadequate for surveillance of adenomas excised by TEM, even if margins are negative and specimens are removed in one piece. With recurrences being identified at varying time frames between 6.4 and 16.9 months, not always on the first surveillance exam, flexible sigmoidoscopy every 6 months for 2 years with full colonoscopy at 3 years—as performed at our institution—does not appear to be excessive, however the optimal frequency is still unclear and further investigation is needed.

Of 36 patients with a final diagnosis of adenocarcinoma, 25 patients declined further treatment and chose surveillance. Of those 4 patients recurred, representing a recurrence rate of 16% in this population of T1 and T2 lesions. Our recurrence rates are similar to that of many studies, which range from 4.7 to 26% depending on T stage^19–22^. Baatrup et al. demonstrated a recurrence rate of 18% for patients with adenocarcinoma treated with TEM. Broken down by T stage, they showed a 13%, 26% and 100% recurrence rate for T1, T2 and T3 lesions, respectively. Mean size in these studies ranged from 2.5 to 3.8 cm, while the frequency of positive margins ranged from 1 to 22%^22^. We further looked at a subpopulation of very low risk lesions, classified as malignant polypectomy scar excisions (n = 11) and T1Sm1 adenocarcinomas with negative margins (n = 9), and noted there were 2/20 recurrences. These lesions were 2.5 cm and 3.5 cm, the latter having high grade tumour budding and focal small vessel lymphovascular invasion. Previous studies have looked at microscopic pathological characteristics of T1 lesions to determine risk factors associated with recurrence. Wirsing et al. demonstrated higher rates of recurrence for Sm2/Sm3 lesions compared with Sm1 (25–60% vs. 14%)^26^. Morino et al. also showed that recurrence after TEM in T1 cancer was dependent on the submucosal depth of invasion, with recurrence rates of 0% for sm1 and recurrence rates of 22.7% for Sm2-3 lesions (which appear to behave more like T2 lesions)^27^. Bach et al. also found increased risk of recurrence with T1 Sm2/3 in addition to identifying intramural lymphovascular invasion as a risk for recurrence^28^. Although T1Sm1 are thought to carry significantly lower recurrence rates, our data suggests that all T1 patients, even T1Sm1, need diligent surveillance and should be scoped frequently as recurrences can occur several years after the original surgery date. Half of the patients who underwent TEM for malignant lesions and suffered local recurrence, were unable to undergo salvage surgery. It is possible the recurrences may have been caught too late. The average timing of recurrences quoted by numerous studies for T1 rectal cancers excised by TEM ranges between 13.0 and 28.7 months, however these same studies have shown recurrences as early as 3 months and as late as 66.5 months^19–21,28^. Similar to the results of our study where the earliest recurrence was caught at 7.4 and the latest was caught at 38.5 months after two previous negative endoscopic exams, this emphasizes the need for early and also continued screening.

The role for surveillance of excised stage I rectal cancers is evolving, and recommendations are in flux, however, there seems to be an awareness of the need for more stringent surveillance in high-risk settings and this includes any tumour excised with local excision. The ASGE guidelines for colonoscopy surveillance after colorectal cancer resection state patients with rectal cancer removed by means of a local excision should be monitored with flexible sigmoidoscopy or EUS every 3–6 months for 2–3 years^29^. ASCRS makes less definitive recommendations for early cancer excised by TEM and other local methods. Their guidelines do recognize the higher potential for recurrence in these patients and the importance of early detection, while acknowledging a shortage of evidence. Their recommendations for endoscopic surveillance of early cancers excised by local excision include proctoscopy every 6 months for 3–5 years and colonoscopy at 1 year^16^. NCCN recommends a surveillance strategy of proctoscopy along with EUS or MRI every 3–6 months for 2 years for locally excised T1 rectal cancers^30^. They also recommend colonoscopy at 1 year^30^. Based on our institution’s data, frequent endoscopy is necessary in the surveillance of rectal cancer excised by TEM, since late detection can result in the inability to salvage. We agree with NCCN and the need for endoscopy every 6 months for 2 years—which is the closest to what is practiced at our institution—but we feel a general consensus on the frequency and length of endoscopic surveillance of the excision site is still lacking.

Limitations of this study include the fact that for many of our patients, post-operative endoscopy was performed outside of the tertiary care teaching hospital and unavailable for data collection. This may contribute to an under-estimation of the recurrences. However, if recurrences may be higher, this only further stresses the need for stringent surveillance. Other limitations include the small study size and the lack of clarity in some charts about the surgeon’s intention for planned surveillance strategies. Nevertheless, we feel even if the intention was not clearly stated up front in the clinic notes, the strategy became clear with the timing of the subsequent endoscopic visits and was therefore deducible. As with any retrospective chart review, our data relies on the comprehensiveness of previously recorded material so not all records were complete with all intended data points. What we would like the reader to understand is that this data is not meant to serve as a basis for creating new guidelines—our numbers are simply much too small. It is meant to shed light on a significant gap in the literature and demonstrate the sizeable range in timeframe of recurrences.

## Conclusion

Our data contributes to the collection of evidence that suggests recurrences of TEM resected lesions, whether benign or malignant, can present early or in a delayed fashion, after several negative endoscopic exams. It suggests a need for an evidence-based guideline for surveillance strategy.

## Data Availability

The datasets generated during the current study are available from the corresponding author on reasonable request.
